# Metastatic lung cancer presenting as thoracic empyema. A Case report

**DOI:** 10.1002/ccr3.2566

**Published:** 2020-02-05

**Authors:** Andrea Leporati, Federico Raveglia, Ugo Cioffi, Matilde De Simone, Filippo Ghelma, Alessandro Baisi

**Affiliations:** ^1^ Thoracic Surgery Unit ASST Santi Paolo e Carlo Università degli Studi di Milano Milano Italy; ^2^ Department of Surgery Università degli Studi di Milano Milano Italy; ^3^ D.I.S.S. – Dipartimento scienze della salute Università degli Studi di Milano Milano Italy

**Keywords:** decortication, preoperative empyema, primary lung cancer, video‐assisted thoracoscopy

## Abstract

Increased cancer risk in patients with inflammatory and infectious diseases has been reported in many studies and lung cancer‐associated empyema in <0.3% patients. We present a patient with empyema in whom the final diagnosis was metastatic lung adenocarcinoma. Purulent pleural fluid obtained by drainage or thoracentesis must always been examined because the association of malignant tumors and empyema should be taken into consideration.

## Graphical abstract

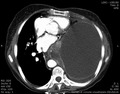

## 1 INTRODUCTION

The incidence of pleural empyema as a primary finding in lung cancer patients is low (0.1%‐0.3%), and only a few references on its management and outcome are reported in the literature.^1^ We describe the case of a patient with empyema in whom the final pathology result was metastatic adenocarcinoma of the lung.

## 2 CASE PRESENTATION

**Figure 1:**
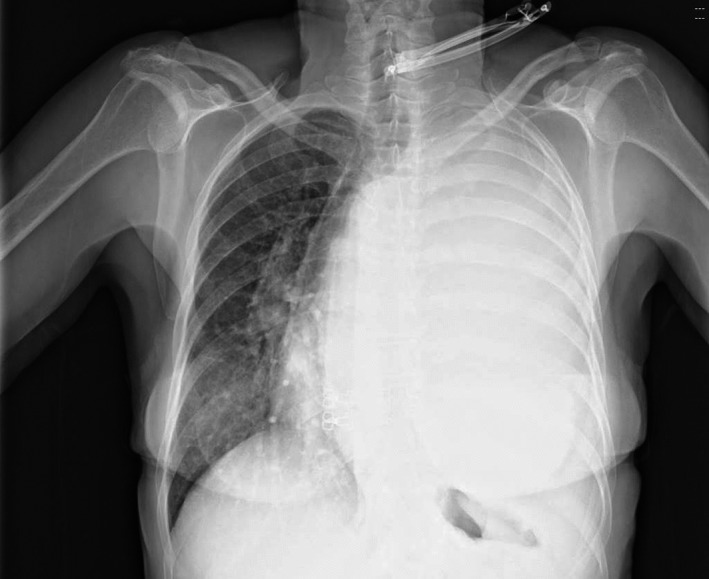
Chest radiography shows a large pleural effusion in the left thorax and mediastinal shifting

**Figure 2:**
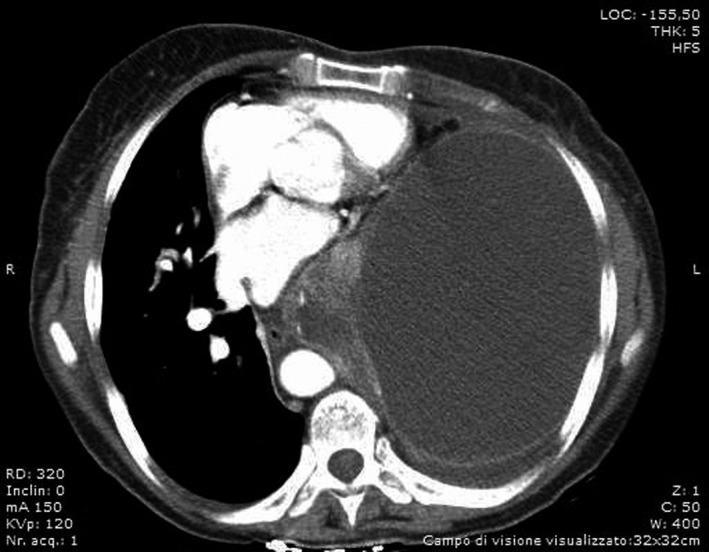
CT scan shows mixed high‐density pleural fluid accumulation in the left and atelectasis of the lung

**Figure 3:**
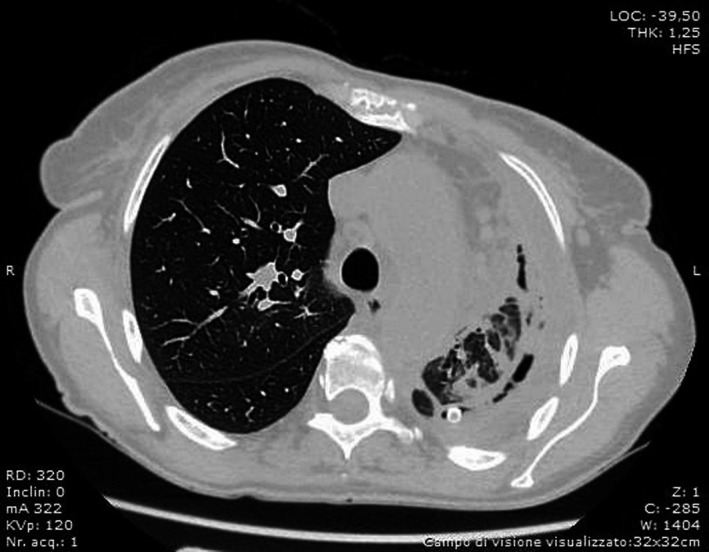
CT scan shows decreasement of pleural effusion with atelectasis of lower left lobe and diffuse pleural thickening

A 59‐year‐old female presented to the emergency department with a 3‐week history of dyspnea, vomiting, and productive cough with one episode of hemoptysis. On admission, blood pressure was 132/80 mm Hg; pulse rate was 106/min, and SaO_2_ was 89%. The patient was apiretic. She smoked 30 cigarettes per day for 40 years. Chest radiographs revealed complete atelectasis of left lung with signs of mediastinal shift (Figure 1). The results of laboratory showed C‐reactive protein (C‐RP) levels of 27.5 mg/dL without leukocytosis and increased transaminase level (alanine aminotransferase [ALT], 0‐38 U/L; aspartate transaminas [AST], 0‐38 U/L. A chest CT scan confirmed a left pleural thickening with mixed high‐density pleural fluid and collapsed lung (Figure 2). A chest tube was placed into the pleural space with evacuation of 1000 cc of dark‐colored purulent liquid. Cytological and microbiological examinations were negative. After thoracic drainage, the respiratory symptoms and vomiting improved, but a daily evacuation of 200/300 cc of pleural fluid persisted. Bronchoalveolar lavage (BAL), performed during flexible bronchoscopy, was negative for malignant cells and showed positive culture for *Klebsiella pneumoniae* and *Staphylococcus aureus*. Patient was treated with amoxicillin and clavulanic acid. Chest CT scan showed reduction of pleural effusion with persistent atelectasis of lower left lobe and diffuse pleural thickening (Figure 3). The patient then subjected to video‐assisted thoracoscopy: At surgery, a massive parietal and visceral pleural thickening was found with the trapped lung. Intraoperative histopathological examination of pleural biopsies showed an acute necrotizing inflammatory background and diffuse inflammation for which thoracoscopy was converted into thoracotomy and lung decortications were performed. Unexpectedly, definitive histology showed pleural metastatic adenocarcinoma of the lung.

## 3 DISCUSSION

Pleural‐thoracic empyema (commonly referred simply as empyema) or pyothorax refers to an infected purulent and often loculated pleural effusion and is the cause of a large unilateral pleural collection. It is a potentially life‐threatening condition requiring prompt diagnosis and treatment.^2^, ^3^ Empyema is usually the complication of another underlying abnormality and may occur in about 5% of cases of pneumonia. Primary lung cancer with associated empyema has been reported in <0.3% patients. In most cases, it is very difficult to obtain a diagnosis of empyema associated with lung cancer before surgery, and often it is a surprise of definitive histology, as in our case.^4^ The prognosis is significantly worsened when pleural metastasis is found.

## 4 CONCLUSION

Physicians must be aware of a possible presence of cancer when patients with empyema are refractory to standard treatment. Studies on pleural fluid including leukocyte counts and cultures, cytological examinations, and neoplasm markers must be planned. Studies on pleural fluid including leukocyte counts and cultures, cytological examinations, and markers of malignancy must be planned.

## CONFLICT OF INTEREST

None declared.

## AUTHORS' CONTRIBUTIONS

All authors carried out the concept and the design of the study and revised the manuscript.

## ETHICS APPROVAL AND CONSENT TO PARTICIPATE

Not applicable.

## CONSENT FOR PUBLICATION

Written informed consent was obtained from the patient for publication of this Case report and any accompanying images. A copy of the written consent is available on request.
